# Association between rs735482 polymorphism and risk of cancer: A meta-analysis of 10 case–control studies

**DOI:** 10.1097/MD.0000000000029318

**Published:** 2022-07-29

**Authors:** Shilin Xue, Wenya Shen, Jianning Cai, Jinhai Jia, Dan Zhao, Shan Zhang, Xiujun Zhao, Ning Ma, Wenjuan Wang, Bingshuang Wang, Xiaolin Zhang, Xuehui Liu

**Affiliations:** a School of Basic Medical Sciences Peking University, Peking University Health Science Center, Beijing, China; b Department of Occupational and Environmental Health, School of Public Health, Hebei Medical University, Hebei Key Laboratory of Environment and Human Health, Shijiazhuang, Hebei, China; c Department of Epidemic Treating and Preventing, Center for Disease Prevention and Control of Shijiazhuang City, Shijiazhuang, China; d Graduate School, Hebei Medical University, Shijiazhuang, China; e Department of Epidemiology and Statistics, School of Public Health, Hebei Medical University, Hebei Province Key Laboratory of Environment and Human Health, Shijiazhuang, Hebei, China.

**Keywords:** cancer, meta-analysis, polymorphism, rs735482, susceptibility

## Abstract

Several studies have inspected the relationship between rs735482 polymorphism and the risk of some human cancers, but the findings remain controversial. We designed this meta-analysis to validate the association between rs735482 polymorphism and cancer risk. All articles were published before September 1, 2018 and searched in Pubmed, Embase, Web of Science, China National Knowledge Infrastructure, WangFang, and Chinese BioMedical databases, STATA 12.0 software was used for statistical analysis, which provides reasonable data and technical support for this article. A total of 10 studies were included in the meta-analysis, including 2652 cancer cases and 3536 rs735482 polymorphic controls. Data were directly extracted from these studies and odds ratios with 95% confidence intervals were computed to estimate the strength of the association. By pooling all eligible studies, the rs735482 polymorphism showed no significant association with susceptibility of several cancers in all the five genetic models (the allelic model: OR = 1.019, 95% CI: 0.916–1.134, *P* = .731). In addition, another adjusted OR data showed a significant increased risk between the rs735482 and susceptibility of several cancers (the codominant model BB vs AA: OR = 1.353, 95% CI: 1.033–1.774, *P* = .028) and the stratification analysis by ethnicity indicated the rs735482 is associated with an increased risk of cancer in Chinese group (BB vs AA, OR = 1.391, 95% CI = 1.054–1.837, *P* = .020; AB+BB vs AA OR = 1.253, 95% CI = 1.011–1.551, *P* = .039). However, the ERCC1 rs735482 is associated with a decreased risk of cancer in Italian group (AB vs AA, OR = 0.600, 95% CI = 0.402–0.859, *P* = .012; AB + BB vs AA, OR = 0.620, 95% CI = 0.424–0.908, *P* = .014). The results of this meta-analysis do not support the association between rs735482 polymorphism and cancer risk. But stratified analysis showed that rs735482 significantly increased the risk of cancer in Chinese while decreased the risk of cancer in Italian. Because of the limited number of samples, larger and well-designed researches are needed to estimate this association in detail.

## 1. Introduction

Cancer is one of the major causes of morbidity and mortality and poses a major threat to public health worldwide. The occurrence of different cancer varies widely among different racial and ethnic groups which may be partly due to the lifestyle and genetic background.^[1]^ The complex interactions of multitudinous gene loci and various environmental factors play an important role in the development of cancer.^[2]^

rs735482 maps in the 3-UTR of excision repair cross complementation group 1 (ERCC1) gene and it also maps in the coding sequence of CD3EAP, a gene that overlaps with ERCC1 but is transcribed in the opposite direction. But their functions are different. The CD3EAP is located between ERCC2 and ERCC1 in the chromosomal region 19q13.3. The CD3EAP products can mediate the activation signal generated by interleukin-2 in T cells.^[3]^ In humans, the CD3EAP has the same part of the coding sequence as ERCC1. This indicated that these genes are linked to some important biological functions.^[4]^ Large population studies have shown that CD3EAP rs735482 polymorphism has a potential impact on the risk of lung cancer and Italics represent significant association with susceptibility of lung cancer.^[5,6]^ CD3EAP rs735482 on Chr19q13.3 were strongly associated with lung cancer risk both in Asian Chinese and in Caucasian Danes.^[7]^

The CD3EAP rs735482 polymorphism results in changes in amino acid 259 (AAA to ACA: K [lysine] to T [threonine]). The missense mutation might affect CD3EAP protein function. ERCC1 located on chromosome 19q13.3^[8–10]^ is an important factor involved in the DNA damage incision, and its products play an important role in nucleotide excision repair (NER). ERCC1 protein, as a highly conserved enzyme, is the main component in the NER process and an essential part of the NER cutting procedure. ERCC1 acts as an endonuclease that incises 5-′ damaged DNA strand, allowing the removal of the damaged strand, polymerization, and relegation.

Epidemiological studies have shown that genetic mutations in ERCC2 and ERCC1 may be associated with various cancer risks.^[11–13]^ Two independent genes, *ERCC1* and *ERCC2*, encode the same mature *ERCC1* sequence.^[14]^ Currently, many studies detected the association between ERCC1 rs735482 polymorphism and risk of various cancers, including breast cancer, cervical cancer, esophageal squamous cell carcinoma, gastric cancer, hepatocellular carcinoma, neuroblastoma, non-small cell lung cancer and other cancers, but the results are still controversial.^[15–23]^ Therefore, we conducted a meta-analysis to find out the effect of rs735482 polymorphism on cancer risk.

## 2. Materials and Methods

### 2.1. Literature search

**Figure 1.**
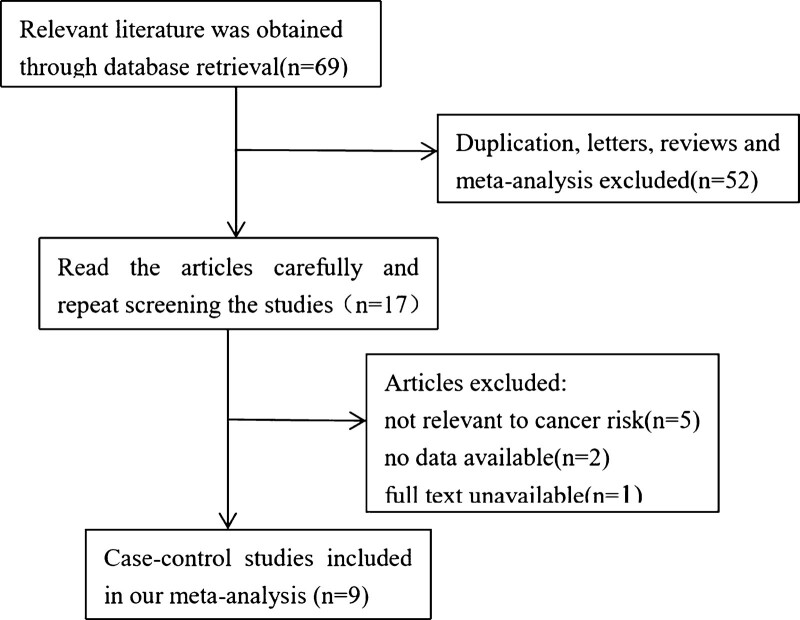
The flow chart illustrates the detailed study selection process of this meta-analysis.

Comprehensive literature searches were conducted for all articles published up to September 30, 2019 in Pubmed, Embase, Web of Science, China National Knowledge Infrastructure, WangFang Date, and Chinese BioMedical databases. These articles described an association between rs735482 polymorphism and cancer risk. The search keywords were “cancer, polymorphism, rs735482.” The process of identifying qualified studies is shown in Figure 1. Criteria for selection of eligible studies included:

(1) the original studies;
(2) studies on the association between rs735482 polymorphism (s) and cancer risk;
(3) studies that reported odds ratio (OR) with 95% confidence interval (CI) values or sufficient data to calculate OR and 95% CI.

The exclusion criteria were:

(1) case-only studies, family-based studies, conference summaries, and review article;
(2) overlapping data;
(3) insufficient genotype information provided. Because this study is a meta-analysis, ethical approval is not necessary.

### 2.2. Data extraction

Two authors searched the literature independently, extracted relevant data, and any disagreement was decided by the third author through discussion. The following data were extracted for each study, including the first author’s name, year of publication, country, ethnicity of participants, number of genotypes in the case–control groups, results of the Hardy–Weinberg equilibrium (HWE) test (Table 1). Another adjusted OR data were extracted for each study including the first author’s name, year of publication, country, the ethnicity of participants, OR and upper and lower limit after correction. The Newcastle-Ottawa Scale was used to evaluate the quality of included studies (Table 2).

**Table 1:** Characteristics of the studies eligible for meta-analysis.

					Case			Control				
Author	Year	Country	Ethnicity	Case/control	AA	AC	CC	AA	AC	CC	HWE	NOS score
Jiaoyang Yin	2013	China	Asian	65/97	10	39	16	22	54	21	0.2635	7
Jiaoyang Yin	2013	China	Asian	330/335	90	163	77	105	167	63	0.8128	7
Tao Yu	2018	China	Asian	300/300	92	150	58	79	160	61	0.2219	7
Nathan R. Jones	2011	America	Caucasian	389/716	289	102	7	523	175	18	0.4652	9
Huang Yu-liang	2018	China	Asian	65/65	20	38	7	28	31	6	0.5334	7
Qianye Zhang	2017	China	Asian	200/200	51	107	42	47	113	40	0.0632	7
Nathan R. Jones	2011	America	Caucasian	160/716	122	37	1	523	175	18	0.4652	9

**Table 2:** Characteristics of the adjusted OR data for meta-analysis.

					Codominant						Dominant			Recessive			
					(AB vs AA)			(BB vs AA)			(AB + BB vs AA)			(BB vs AA + AB)			
Author	Year	Country	Ethnicity	Case/control	OR	Up	Low	OR	Up	Low	OR	Up	Low	OR	Up	Low	NOS score
Jiaoyang Yin	2016	China	Asian	330/335	1.28	0.89	1.85	1.66	1.06	2.62	1.38	0.98	1.96	1.42	0.97	2.09	7
Fulvio Ricceri	2009	Italy	Italian	324/283	0.6	0.4	0.89	0.82	0.25	2.67	0.62	0.42	0.9	0.94	0.29	3.03	8
Jiaoyang Yin	2015	China	Asian	489/489	1.15	0.86	1.54	1.25	0.88	1.78	1.18	0.9	1.55	1.15	0.85	1.55	7

### 2.3. Statistical analysis

Meta-analysis was performed using STATA 12.0 software. HWE for each study was determined by the chi-square test to verify the representativeness of the study population. If *P* < .05, the genotypes of the control group were inconsistent with HWE. The relationship between rs735482 polymorphism and cancer risk was estimated by ORs and 95% CIs. Pooled ORs and 95% CIs for codominant (AC vs AA and CC vs AA), dominant (AC + CC vs AA), recessive (CC vs AC + AA), and the allelic (C vs A) genetic models were estimated. As for adjusted OR data, pooled ORs and 95% CIs for codominant (AB vs AA and BB vs AA), dominant (AB + BB vs AA), recessive (BB vs AB + AA) genetic models were estimated. *Z* test was used to evaluate the significance of the pooled OR, and *P* < .05 was considered statistically significant. The results of the heterogeneity test were used to determine whether the fixed effect model or the random effect model. The heterogeneity test was measured by *Q* test and *I*^2^ statistics. If the test result is *I*^2^ ≥ 50% or *P* < .1, indicating the presence of heterogeneity, the random effect model is selected. Otherwise, the fixed-effects model is adopted. Begg’s funnel plots were conducted under all genetic models to assess the publication bias, and the asymmetric plots implied potential publication bias. The degree of asymmetry was measured using Egger’s test and *P* < .1 was considered a significant publication bias. The robustness was tested by leave-one-out sensitivity analyses.

## 3. Results

### 3.1. Study characteristics

Our meta-analysis included 10 case–control studies that met the inclusion criteria, including 2652 cancer cases and 3536 controls. Tables 1 and 2 show the characteristics and relevant data of the included studies.

### 3.2. Main analysis results

**Figure 2.**
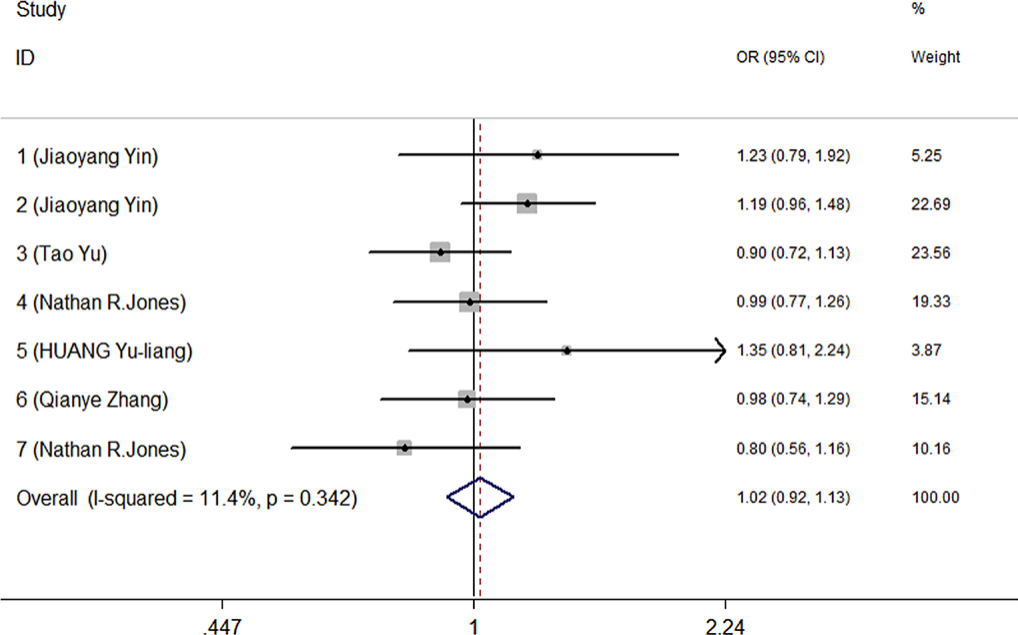
The forest plot for the relationship between ERCC1 rs735482 polymorphism and cancer susceptibility in the allelic model (C vs A).

**Figure 3.**
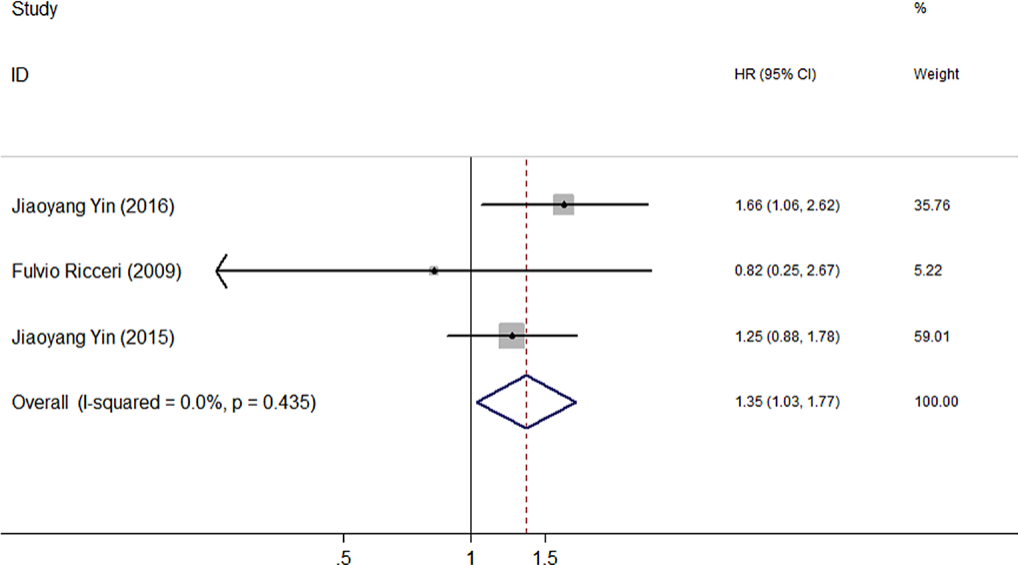
The forest plot of the adjusted OR data for the relationship between ERCC1 rs735482 polymorphism and cancer susceptibility in the codominant model (BB vs AA).

According to the results of the meta-analysis of 10 eligible studies, it was found that there was no correlation between rs735482 polymorphism and cancer risk of the general population in the codominant, dominant, recessive and allele model test (Fig. 2 and Table 3). Another adjusted OR data showed a significantly increased risk between rs735482 and several types of cancer susceptibility in the codominant model (BB vs AA) test (Fig. 3 and Table 4).

**Table 3:** The pooled ORs and 95% CIs for the association between ERCC1 rs735482A>C polymorphism and cancer susceptibility.

	Association			Heterogeneity			Publication bias	
Genetic model	OR (95% CI)	z	*P*	?^2^	*P*	*I*^2^ (%)	Begg’s (*P*)	Egger’s (*P*)
AC vs AA	1.014 (0.868–1.184)	0.18	.859	5.6	.469	0	.548	.268
CC vs AA	1.036 (0.810–1.325)	0.28	.779	7.3	.294	17.8	.548	.57
CC + AC vs AA	1.012 (0.872–1.174)	0.15	.878	7.01	.32	14.4	.548	.287
CC vs AA + AC	1.052 (0.850–1.300)	0.46	.643	4.67	.587	0	.23	.151
C vs A	1.019 (0.916–1.134)	0.34	.731	6.78	.342	11.4	1	.681

**Table 4:** The pooled ORs and 95% CIs for the association between ERCC1 rs735482A>C polymorphism and cancer susceptibility of the adjusted OR data.

	Association			Heterogeneity			Publication bias	
Genetic model	OR (95% CI)	z	*P*	?^2^	*P*	*I*^2^ (%)	Begg’s (*P*)	Egger’s (*P*)
AB vs AA	0.972 (0.634–1.489)	0.12	.908	8.89	.012	77.5	1	.57
BB vs AA	1.353 (1.033–1.774)	2.19	.028	1.67	.435	0	1	.708
BB + AB vs AA	1.014 (0.656–1.570)	0.06	.949	10.43	.005	80.8	1	.611
BB vs AA + AB	1.232 (0.977–1.554)	1.77	.077	0.93	.627	0	1	.835

### 3.3. Subgroup analysis results

**Figure 4.**
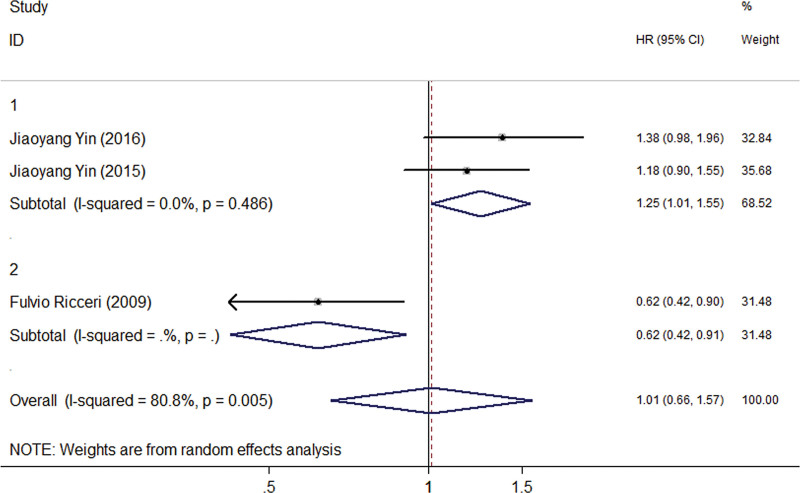
The forest plot of the stratified analysis for the relationship between ERCC1 rs735482 polymorphism and cancer susceptibility in the dominant model (AB + BB vs AA).

Stratified analysis of rs735482 polymorphism was done by ethnicity (Table 5). The data suggested that rs735482 was associated with an increased risk of cancers in the Chinese group (BB vs AA, OR = 1.391, 95% CI = 1.054–1.837, *P* = .020; AB + BB vs AA, OR = 1.253, 95% CI = 1.011–1.551, *P* = .039). However, rs735482 was associated with a decreased risk of cancers in Italian group (AB vs AA, OR = 0.600, 95% CI = 0.402–0.859, *P* = .012; AB + BB vs AA, OR = 0.620, 95% CI = 0.424–0.908, *P* = .014) (Fig. 4).

**Table 5:** Stratified analysis of ERCC1 rs735482A>C variant on cancer susceptibility.

		Dominant (BB + AB vs AA)			Recessive (BB vs AA + AB)			Homozygous (BB vs AA)			Heterozygous (AB vs AA)		
Subgroup	N	OR (95% CI)	*I*^2^ (%)	*P*-value	OR (95% CI)	*I*^2^ (%)	*P*-value	OR (95% CI)	*I*^2^ (%)	*P*-value	OR (95% CI)	*I*^2^ (%)	*P*-value
Total	3	1.104 (0.656–1.570)	80.8	.949	1.232 (0.977–1.554)	0	.077	1.353 (1.033–1.774)	0	.028	0.972 (0.634–1.489)	77.5	.895
Chinese	2	1.253 (1.011–1.551)	0	.039	1.246 (0.983–1.578)	0	.068	1.391 (1.054–1.837)	0	.02	1.199 (0.954–1.506)	0	.119
Italian	1	0.62 (0.424–0.908)	0	.014	0.940 (0.291–3.038)	0	.918	0.820 (0.251–2.680)	0	.743	0.60 (0.402–0.895)	0	.012

#### 3.3.1. Heterogeneity and publication bias.

**Figure 5.**
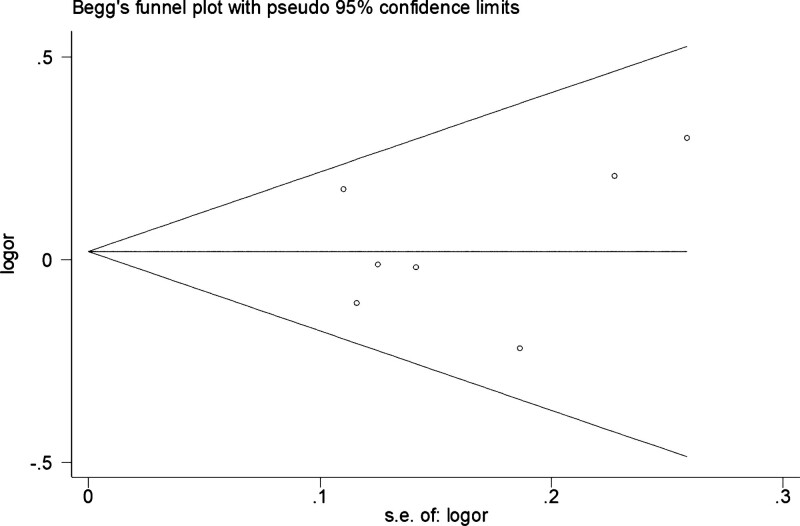
The funnel plot for the test of publication bias in the recessive model (CC vs AC + AA).

The heterogeneity of meta-analysis results is shown in Tables 2 and 3. The results showed that some studies had heterogeneity. The potential publication bias was estimated by Begg’s funnel plot and Egger’s test. As shown in Figure 5, the shape of funnel plots was symmetrical and no publication bias was observed.

#### 3.3.2. Sensitivity analysis.

**Figure 6.**
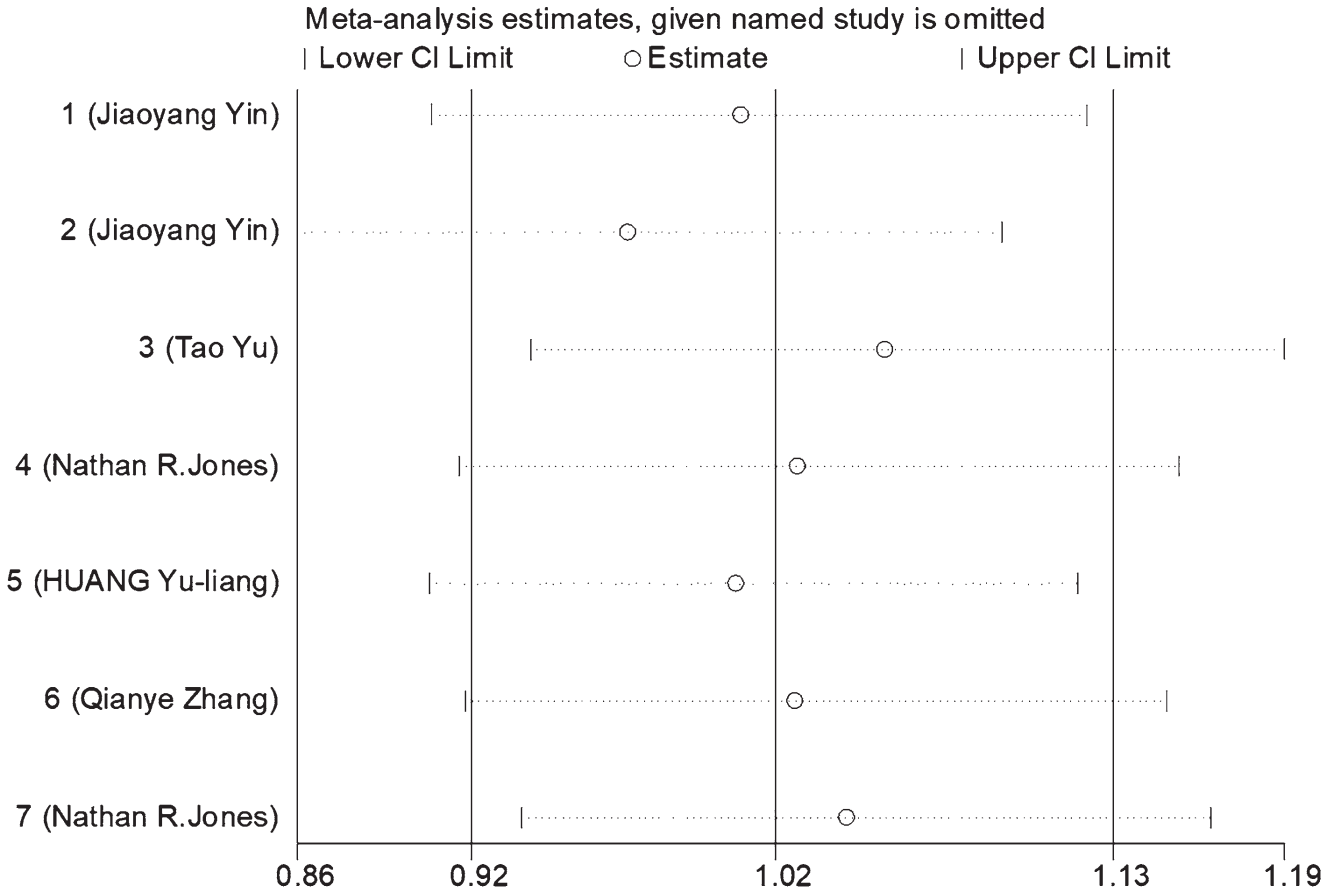
Sensitivity analyses for studies between ERCC1 rs735482 polymorphism and cancer susceptibility in the allelic model (C vs A).

**Figure 7.**
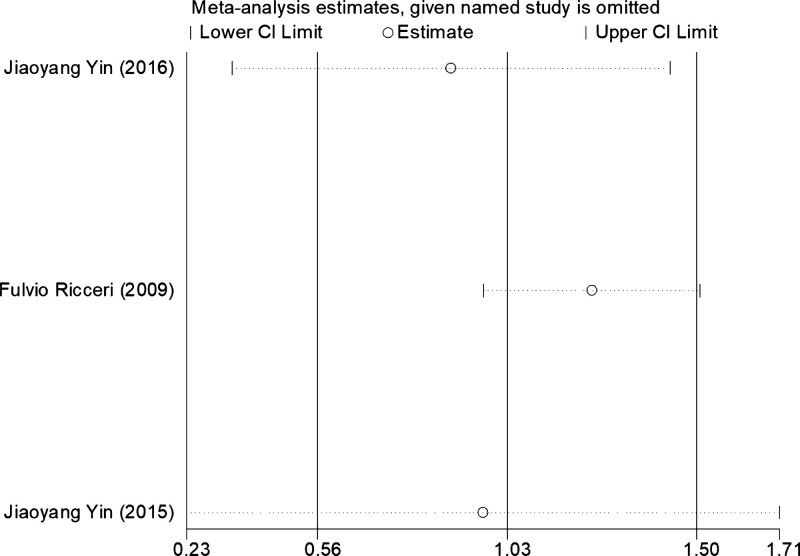
Sensitivity analyses of the adjusted OR data between ERCC1 rs735482 polymorphism and cancer susceptibility in the dominant model (AB + BB vs AA).

A sensitivity analysis was performed to assess the impact of a particular publication on the overall assessment. The results of the relevant pooled ORs showed that there was no significant change when each study was neglected (Fig. 6). Thus, the final pooled results were both stable and reliable. But the relevant pooled ORs of the adjusted OR showed that significant change occurred when each study was neglected, one at a time, from the meta-analysis in dominant (BB + AB vs AA) and codominant (AB vs AA) genetic models (Fig. 7).

## 4. Discussion

A variety of studies have been conducted to determine whether rs735482 polymorphism affects the susceptibility of several types of cancer, and these findings have been controversial. In this meta-analysis, a total of 10 eligible case–control studies were included and their combined results were used to assess the relationship between rs735482 polymorphism and cancer susceptibility. Our meta-analysis showed that the rs735482 polymorphism had no significant effect on cancer risk. While the adjusted OR data showed a significantly increased risk between the rs735482 and susceptibility of several cancers (the codominant model BB vs AA, OR = 1.353, 95% CI: 1.033–1.774, *P* = .028), and the stratification analysis by ethnicity indicated the rs735482 was associated with an increased risk of cancers in Chinese group (BB vs AA, OR = 1.391, 95% CI = 1.054–1.837, *P* = .020; AB + BB vs AA, OR = 1.253, 95% CI = 1.011–1.551, *P* = .039) and a decreased risk of cancers in Italian group (AB vs AA, OR = 0.600, 95% CI = 0.402–0.859, *P* = .012; AB + BB vs AA, OR = 0.620, 95% CI = 0.424–0.908, *P* = .014).

In the stratified analysis of the adjusted OR data by ethnicity, we found that individuals carrying the variant C allele could increase the cancer susceptibility in Asians, but reduce cancer susceptibility of Italians, suggesting that racial differences might play an important role in genetic background variation.^[24–26]^

CD3EAP is a complementary nucleoprotein to ERCC1 and can participate in cell proliferation as part of the RNA polymerase I transcription complex.

Many studies have been published on the possible association between ERCC1 polymorphisms and cancers.^[7,27–29]^ However, the most important studies that ERCC1 polymorphisms may be involved in are the functional studies relating to ERCC1 gene variant, repairability, and gene expression levels. All these results strongly support a possible role of ERCC1 in cancer risk. The ERCC1 rs735482 is located in the chromosomal region19q13.3, and haplotypes of chromosome 19q13.2–3 have been associated with cancer risk in previous studies. *ERCC1* rs735482 is involved in the identification and removing platinum-induced intra-strand adducts in DNA, and is resistant to platinum-based chemotherapy in many cancers, including non-small cell lung cancer, ovarian cancer, bone tumor, and colorectal cancer.^[13,28–31]^

There are several limitations in our meta-analysis. Firstly, there is heterogeneity between some studies. Heterogeneity can be attributed to differences in ethnicity, source of control, status, and type of cancer. Secondly, the language of the study is limited to English. Thirdly, although the combination of genetic factors and environmental exposure was taken into account in the adjusted OR, the number of studies and the sample size were small. Fourthly, different genotyping methods may lead to bias in the analysis. Finally, our meta-analysis was limited to Asian, Caucasian, Italian population and lack of other ethnicities.

To sum up, our meta-analysis showed that rs735482 polymorphism was not a risk factor for cancer, but the pooled adjusted OR showed that there was an association between the rs735482 polymorphism and susceptibility of several cancers, and the stratification analysis by ethnicity indicated the rs735482 was associated with an increased risk of cancer in Chinese while a decreased risk of cancer in Italian. To clarify the possible role of this mutation in cancer, further well-designed studies with a wide ethnic background, detailed biological characteristics, and larger samples number are required.

## Acknowledgments

We greatly acknowledge Tao Yu,^[32]^ Qianye Zhang,^[21]^ and Jiaoyang Yin,^[33,34]^ for they have provided the frequencies of rs735482 C/A polymorphism between the cases and controls in detail.

## Author contributions

Shilin Xue, Wenya Shen, and Jianning Cai wrote the main manuscript text and prepared all figures and tables. All authors reviewed the manuscript.

Data curation: Bingshuang Wang, Dan Zhao.

Funding acquisition: Xiaolin Zhang.

Methodology: Shan Zhang.

Software: Jinhai Jia.

Supervision: Xiujun Zhao.

Validation: Ning Ma, Wenjuan Wang.

Writing – original draft: Wenya Shen, Xuehui Liu.

Writing – review & editing: Fengxue Yu, Jianning Cai, Shilin Xue.
